# An Experimental Medicine Investigation of the Effects of Subacute Pramipexole Treatment on Emotional Information Processing in Healthy Volunteers

**DOI:** 10.3390/ph14080800

**Published:** 2021-08-14

**Authors:** Marieke Annie Gerdine Martens, Alexander Kaltenboeck, Don Chamith Halahakoon, Michael Browning, Philip J. Cowen, Catherine J. Harmer

**Affiliations:** 1 Department of Psychiatry, University of Oxford, Warneford Hospital, Oxford OX3 7JX, UK; alexander.kaltenboeck@meduniwien.ac.at (A.K.); don.halahakoon@msdtc.ox.ac.uk (D.C.H.); michael.browning@psych.ox.ac.uk (M.B.); phil.cowen@psych.ox.ac.uk (P.J.C.); catherine.harmer@psych.ox.ac.uk (C.J.H.); 2 Wellcome Centre for Integrative Neuroimaging, University of Oxford, Oxford OX3 7JX, UK; 3 Clinical Division of Social Psychiatry, Department of Psychiatry and Psychotherapy, Medical University of Vienna, Vienna General Hospital, 1090 Vienna, Austria; 4 Oxford Health NHS Foundation Trust, Warneford Hospital, Oxford OX3 7JX, UK

**Keywords:** pramipexole, dopamine, emotion, emotional information processing, affective information processing, depression, antidepressant, neuroimaging

## Abstract

Treatment with the dopamine D2/D3 receptor agonist pramipexole has demonstrated promising clinical effects in patients with depression. However, the mechanisms through which pramipexole might alleviate depressive symptoms are currently not well understood. Conventional antidepressant drugs are thought to work by biasing the processing of emotional information in favour of positive relative to negative appraisal. In this study, we used an established experimental medicine assay to explore whether pramipexole treatment might have a similar effect. Employing a double-blind, parallel-group design, 40 healthy volunteers (aged 18 to 43 years, 50% female) were randomly allocated to 12 to 15 days of treatment with either pramipexole (at a peak daily dose of 1.0 mg pramipexole salt) or placebo. After treatment was established, emotional information processing was assessed on the neural level by measuring amygdala activity in response to positive and negative facial emotional expressions, using functional magnetic resonance imaging (MRI). In addition, behavioural measures of emotional information processing were collected at baseline and on drug, using an established computerized task battery, tapping into different cognitive domains. As predicted, pramipexole-treated participants, compared to those receiving placebo, showed decreased neural activity in response to negative (fearful) vs. positive (happy) facial expressions in bilateral amygdala. Contrary to our predictions, however, pramipexole treatment had no significant antidepressant-like effect on behavioural measures of emotional processing. This study provides the first experimental evidence that subacute pramipexole treatment in healthy volunteers modifies neural responses to emotional information in a manner that resembles the effects of conventional antidepressant drugs.

## 1. Introduction

Depression is considered a leading cause of disability worldwide. Although various medications have shown clinical efficacy in this condition [1], a considerable number of patients do not experience sufficient symptom improvement following conventional antidepressant treatment, even when multiple therapeutic attempts are made [2]. Hence, it is vital for psychopharmacological research to continue exploring potential new antidepressant treatment options.

To date, commonly used drugs for the treatment of depression primarily target serotonin and/or norepinephrine transmission [1,2]. However, accumulating evidence from animal and human research suggests that pharmacological agents targeting specifically the dopamine system might also constitute useful treatments for depression [3,4].

One agent that has received attention in this specific context recently is the non-ergot dopamine agonist pramipexole [2,5]. This drug shows selective affinity for and full intrinsic activity at the dopamine D2 receptor subfamily, particularly the D3 receptor, which is found in high concentration in mesolimbic areas implicated in mental processes related to emotion and mood [5,6]. In a recent meta-analysis, including 504 patients with major depressive episodes in total, it was found that pramipexole treatment was associated in the short-term with a response rate of 52.2% and a remission rate of 36.1% [5]. Focusing on the long-term effects, these numbers increased to a 62.1% response rate and a 39.6% remission rate [5]. Furthermore, in randomised clinical trials, pramipexole treatment was associated with a superior clinical response rate relative to placebo and a similar response rate compared to selective serotonin reuptake inhibitors [5].

Whilst several studies have demonstrated clinical efficacy of pramipexole treatment in depression, it is currently not known through which mechanisms the drug might bring about its antidepressant effects. Different mechanisms on different levels of neuroscientific description have been proposed, including activation and increased expression of dopamine receptors of the D2 subfamily [7,8], dopamine autoreceptor and dopamine transporter interaction [9], inhibition of NMDA receptors and/or NO-cGMP synthesis [7], increase in dopamine and serotonin neurotransmission [10], anti-inflammatory effects [11], changes in metabolism in certain brain regions [12], and inhibition of aversive information processing [13].

A contemporary mechanistic framework to explain treatment effects of conventional antidepressant agents (e.g., SSRIs) is the so-called “cognitive neuropsychological model of antidepressant treatment action” (see for example [14,15]). This model suggests that efficacious antidepressant treatments, regardless of their idiosyncratic molecular target profiles, exert their clinical effects via a shared ability to (sub-) acutely shift the processing of emotional information towards a preference for positive relative to negative input. This induced bias in favour of positive emotional material is thought to counterbalance the negatively biased emotional information processing that is typically found in patients with depression and that is thought to elicit and maintain a depressive episode.

The cognitive theory is well-supported by a growing corpus of literature. The induction of positive biases in emotional information processing has been documented for various antidepressant agents, both on the neural as well as on the behavioural level of description. For example, different antidepressant drugs (e.g., citalopram, reboxetine, mirtazapine, bupropion) have been shown to increase recognition of positive facial expressions, decrease recognition of negative facial expressions, fasten responses for categorization of positive relative to negative emotional words, or heighten recall of positive versus negative emotional words following single or subacute dosing [15,16]. Similar valence-dependent effects have also been documented on the neural level, specifically with regard to amygdala activity. Here too, different antidepressant treatments (e.g., citalopram, reboxetine, mirtazapine) have been shown to increase amygdala activity in response to positive emotional material and/or decrease amygdala activity in response to negative emotional material [15,17].

Importantly, valence-dependent neural and behavioural effects can be observed both in patients with depression as well as in healthy volunteers, suggesting that they are an inherent effect of antidepressant treatment and not simply an epiphenomenon of subtle improvements in psychopathology [15,17]. Moreover, effects on emotional information processing can be measured long before improvements in the clinical state normally become apparent in patients with depression and these effects are associated with later occurring clinical improvements [18,19,20]. Based on these observations, it could recently be shown that an assessment of early effects on emotional processing in patients undergoing antidepressant treatment in primary care can be effectively used to guide subsequent treatment choices and improve certain clinical outcomes [21].

It is currently not known whether pramipexole has effects on emotional information processing that are comparable to those of conventional antidepressant drugs. We, therefore, studied emotional information processing following subacute pramipexole treatment using an established healthy volunteer assay. Forty healthy volunteers were randomly assigned to placebo (*n* = 19) or pramipexole (*n* = 21), titrated up to a dose of 1.0 mg (of pramipexole salt) over ten days. Participants stayed on this dose until all on-drug testing was finished, which was (at the latest) day 15 of treatment. Participants completed a neuroimaging assessment of amygdala response to positive and negative emotional material between day 12 and day 15. For this, an established emotional faces task was used, in which participants are shown happy (positive) and fearful (negative) emotional faces, which are known to reliably elicit amygdala activity [22]. Participants also completed a behavioural assessment of emotional bias at baseline and between treatment days 12 to 15. For this, the Oxford Emotional Test Battery, an established set of computerized tasks tapping into emotional information processing in different cognitive domains, was used.

We hypothesised that subacute pramipexole treatment would alter emotional information processing both on the neural and on the behavioural level comparable to conventional antidepressant drugs. Thus, we predicted that pramipexole would attenuate amygdala response to negative emotional stimuli and/or increase the response to positive emotional stimuli. Furthermore, we expected that pramipexole would induce a positive behavioural bias in emotional information processing across cognitive domains.

## 2. Results

### 2.1. Study Sample

The treatment groups were well-matched for basic demographic, physical, and psychological characteristics; also see Table 1. Two participants (both receiving placebo) dropped out of the study before on-drug assessments were conducted because of subjective side effects. They were subsequently replaced by new volunteers. 

### 2.2. What Were the Side Effects of Pramipexole and Was Blinding Successful?

Self-reported side effects significantly differed between groups only with regards to nausea. In the pramipexole group, participants reported nausea significantly more often than in the placebo group (61.9% vs. 5.3%, Fisher’s exact test: *p* < 0.001); also see Table 2. 

Blinding of volunteers and researchers was not fully achieved. Study participants who received pramipexole correctly guessed their treatment allocation in 61.9% of cases, and those receiving placebo correctly guessed so in 84.2% of cases (Fisher’s exact test: *p* = 0.004). The researchers were able to correctly guess the administered treatment in 71.4% of all pramipexole-treated participants and in 84.2% of placebo-treated study participants (Fisher’s exact test: *p* = 0.001).

### 2.3. Did Pramipexole Treatment Influence Subjective State?

Pramipexole treatment did not alter measures of subjective state (F ≤ 3.1, *p* ≥ 0.08); also see Table 3.

### 2.4. Did Pramipexole Treatment Induce a Positive Bias in Behavioural Measures of Emotional Information Processing?

#### 2.4.1. Facial Expression Recognition Task (FERT)

One participant was excluded from the FERT analysis because they did not classify any of the fearful faces correctly during the on-drug study visit, which suggests that they might have misunderstood the instructions or did not engage with the task.

No significant effects of treatment (i.e., group × visit interaction or group × visit × emotion interaction) were found for any of the task outcomes: hit rate (i.e., percentage of stimuli correctly labelled), F ≤ 0.3, *p* ≥ 0.57; false alarm rate (i.e., percentage of stimuli labelled with an incorrect emotion), F ≤ 0.3, *p* ≥ 0.62; non-identification rate (i.e., percentage of emotional stimuli incorrectly labelled as having a neutral expression), F ≤ 0.3, *p* ≥ 0.62; and reaction time, F ≤ 0.3, *p* ≥ 0.78. There was also no significant treatment effect on neutral face hit rate (F (1.35) = 2.5, *p* = 0.12, one outlier excluded) or reaction time (F (1.36) = 0.1, *p* = 0.80), see Table 4. To complement the analysis, we also used an alternative approach comparing groups on signal detection theory measures (i.e., target sensitivity and response bias, calculated following Grier [23]). This analysis also showed no significant effect of pramipexole treatment on facial expression recognition.

#### 2.4.2. Emotional Categorisation Task (ECAT)

Engagement in the task was appropriate for all participants at each visit (minimum accuracy ≥ 82.1%). Analysis of reaction time showed no significant treatment effect (i.e., group × visit interaction or group × visit × valence interaction), F ≤ 2.1, *p* ≥ 0.16, Table 4. 

#### 2.4.3. Faces Dot Probe Task (FDOT)

Engagement in the task was appropriate for all participants at each visit (minimum accuracy ≥ 85.4%). Analyses of the outcome measure vigilance bias score were conducted separately for the masked and unmasked conditions. In the masked condition, there was no significant treatment effect (i.e., group × visit interaction or group × visit × valence interaction), F ≤ 0.3, *p* ≥ 0.61. The same pattern was observed in the unmasked condition, F ≤ 2.0, *p* ≥ 0.16, Table 4. 

#### 2.4.4. Emotional Recall Task (EREC)

With regards to the number of hits (i.e., words correctly recalled), there were no significant effects of treatment (i.e., group × visit interaction or group × visit × valence interaction), F ≤ 1.0, *p* ≥ 0.31. The same pattern was observed with regards to intrusions (i.e., number of incorrectly recalled words), F ≤ 2.9, *p* ≥ 0.1. Numerically, the pramipexole group showed a greater increase in incorrectly recalled words independent of valence, Table 4.

### 2.5. Did Pramipexole Treatment Affect Neural Measures of Emotional Information Processing?

In order to determine if the functional MRI (fMRI) task engaged brain regions previously associated with fearful and happy facial stimuli, blood-oxygen-level-dependent (BOLD) activation in response to fearful faces, happy faces, and the mean of both valences was compared to the baseline (i.e., fixation cross) across both groups. Significant brain activations were observed in a network of areas that match previous reports [22,24,25]. These findings, therefore confirm that the task engaged brain regions that are part of a network implicated in the processing of both fear and happiness. See also Supplementary Figure S1. 

#### 2.5.1. Effect of Treatment on Amygdala Activity (Region of Interest Analysis)

**Figure 1:**
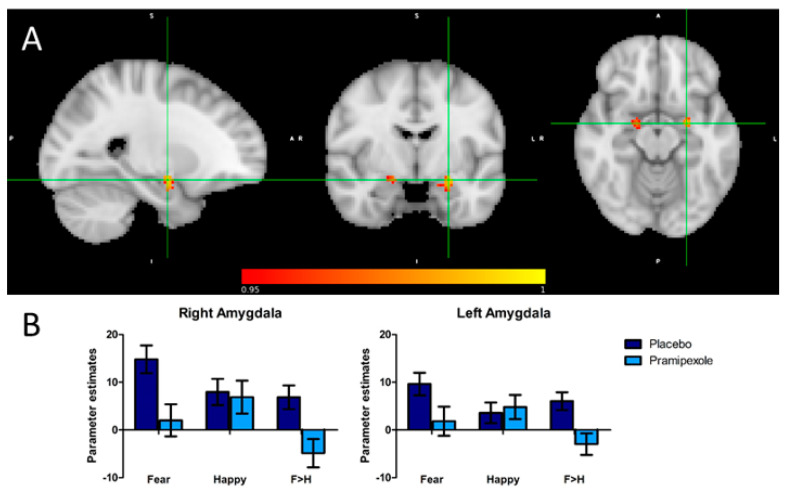
(**A**) Sagittal, coronal and axial images depicting significantly reduced activation in the pramipexole relative to the placebo group for the fear versus happiness contrast in the left and right amygdala. Peak voxels right amygdala: x = 18, y   =   −6, z   =   −14; voxel size: 35; 1 − *p* = 0.979. Peak voxels left amygdala: x = −24, y = −6, z = -14; voxel size = 83; 1 − *p* = 0.994. Results are shown TFCE-corrected with a family-wise error cluster significance level of 1 − *p* > 0.95. (**B**) Parameter estimates extracted from the significant clusters in response to fearful versus happy faces. Error bars represent the standard error of the mean.

To establish the effect of pramipexole treatment on amygdala activity specifically related to emotional processing, BOLD activity in response to fearful (i.e., negative) versus happy (i.e., positive) facial expressions was investigated. In both the left amygdala (voxel size: 83, peak voxel location: x = −24, y = −6, z = −14, *p* = 0.006) and the right amygdala (voxel size: 35, peak voxel location: x = 18, y = −6, z = −14, *p* = 0.02), there was reduced BOLD activity in the pramipexole relative to the placebo group in response to fearful versus happy facial expressions (see Figure 1).

#### 2.5.2. Analyses Controlling for Potential Confounds

##### Arterial Spin Labelling (ASL)—Effect of Treatment on Whole Brain Perfusion

Interpreting differences in neural activity measured by BOLD fMRI can be complicated by between-group variation in total or regional brain perfusion [26]. Previous studies have found effects of dopaminergic drug treatment on resting cerebral perfusion [27,28]—therefore, resting perfusion maps (ASL-data) were added as voxel-dependent explanatory variables of no interest in the fMRI task analyses to account for potential differences in perfusion between the groups. When the groups were explicitly compared, however, there were no significant differences in whole brain voxel-wise regional perfusion (placebo > pramipexole: *p* = 0.625, pramipexole > placebo: *p* = 0.170) nor global grey matter perfusion (t(38) = -0.47, *p* = 0.55), placebo: M = 61.0 mL/100 g/min, SD = 8.0; pramipexole: M = 62.3 mL/100g/min SD = 9.1.

##### Effect of Treatment on Amygdala Perfusion (Region of Interest Analysis)

Defining the amygdala as an region of interest (ROI) (similar to task fMRI analysis), no differences in voxel-wise perfusion were found either (left amygdala, placebo > pramipexole: *p* = 0.292, pramipexole > placebo: *p* = 0.402; right amygdala, placebo > pramipexole: *p* = 0.470, pramipexole > placebo: *p* = 0.436).

#### 2.5.3. Behavioural Performance in the fMRI Task

There were significant main effects of emotion across groups, with participants being more accurate and faster at identifying the gender of happy faces than that of fearful faces (F (1.38) = 26.5, *p* < 0.001; F (1.38) = 5.6, *p* = 0.02). There were, however, no main effects of treatment (F (1.38) = 1.2, *p* = 0.29; F (1.38) = 2.3, *p* = 0.14), or treatment × emotion interactions (F (1.38) = 0.02, *p* = 0.88; F (1.38) = 1.0, *p* = 0.32) in either measure.

## 3. Discussion

This study explored the effects of subacute treatment with the dopamine D2 receptor agonist pramipexole on neural and behavioural measures of emotional information processing. In line with our predictions, and a contemporary cognitive neuropsychological model of antidepressant action, pramipexole treatment led to attenuated amygdala activity in response to negative (fearful) relative to positive (happy) facial expressions. However, contrary to our predictions, there was no effect of pramipexole on behavioural measures of emotional information processing.

The observed influence of pramipexole on neural responses to negative (fearful) facial expressions parallels the effects of conventional antidepressant drugs in healthy study participants [15,17]. The ability to dampen amygdala response to negative emotional material has also been demonstrated previously, for example, for citalopram, reboxetine, or mirtazapine [15]. The cognitive model assumes that this effect constitutes a crucial common mechanism through which antidepressant efficacy is brought about and our data, therefore, suggest that pramipexole’s antidepressant effect could also be explained by this framework.

Interestingly, most drugs that have been studied with regards to their effects on emotional information processing target serotonergic and/or noradrenergic neurotransmission [15]. Pramipexole, however, is a selective agonist at the dopamine D2 receptor subfamily with little to no affinity for serotonergic or noradrenergic receptors [6]. The valence-specific effects on amygdala activity reported here could therefore also be interpreted as evidence that a shift in emotional information processing towards positivity can be achieved not only by potentiating serotonin or norepinephrine function but also by targeting dopaminergic neurotransmission. In support of this idea, single-dose treatment with the dopamine D2 receptor antagonist sulpiride has also been reported to selectively decrease recognition for angry faces in healthy participants [29], an effect that resembles that of the established antidepressant fluoxetine [30].

An alternative explanation, however, might be that by stimulating dopamine D2 receptors, pramipexole treatment gives rise to downstream changes in serotonin and/or norepinephrine signalling, which in turn leads to antidepressant-like effects on emotional information processing. Supporting this idea, it has previously been shown that pramipexole administration can indeed also affect serotonergic neurotransmission [10,31].

Further research will be required to elucidate which of these interpretations is correct. Yet, the specific neurochemical underpinnings notwithstanding, our results indicate that pramipexole has neurocognitive effects that are opposite to those observed in patients with depression [32] and that resemble the effects of conventional antidepressant treatments [14,15]. Hence, our data provide further (mechanistic) support to the idea that pramipexole might be a useful antidepressant intervention.

Interestingly, the valence-specific effect we observed on the neural level did not translate into a more positively biased behavioural performance in the Oxford Emotional Test Battery. There are several potential explanations for this discrepancy in neural and behavioural effects. 

One possibility is that the neural measures we studied are more sensitive to pramipexole treatment than the behavioural tasks, and hence the study might have lacked the power to detect effects in the latter. We only used a moderately-sized sample and only employed subacute treatment for up to 15 days (which is still relatively short compared to long-term pramipexole treatment used clinically). Moreover, although we administered a comparatively high dose for a healthy volunteer study, our target dose (1.0 mg pramipexole salt) was still relatively low compared to doses commonly used in depression [5,33]. Hence, a larger sample size or a more intensive treatment regimen might have been required to also uncover behavioural effects.

Alternatively, practice effects might have obscured valence-specific effects in task performance, as participants might have learned how to respond to stimuli efficiently during the first visit. Given that there was no baseline assessment for the neural measures, this practice effect would have manifested only in the behavioural tasks.

Of note, pramipexole had no effect on subjective state measures. This is comparable to other antidepressant interventions, which generally also do not show such effects in healthy volunteers [16,25,34].

We also found no effect of subacute pramipexole treatment on resting brain perfusion. This is in contrast with a previous study that investigated acute effects of pramipexole (0.5 mg dose) on cerebral perfusion at rest and found enhanced blood flow in the nigrostriatal system (brainstem at the level of the substantia nigra and caudate nucleus) and reduced cerebral blood flow in the thalamus, visual cortex, and cerebellum [28]. Further studies comparing different treatment regimens with pramipexole (e.g., acute versus subacute) and varying doses (e.g., low-dose versus high-dose) are needed in the future to better understand these divergent results.

The results reported here should be interpreted in the light of several strengths and limitations pertaining to our study.

To our knowledge, this is the first published investigation of the potential effects of subacute pramipexole treatment on emotional information processing. These results add to a growing corpus of literature probing the valence-specific effects of conventional and potential novel antidepressant interventions on neural and behavioural measures of emotional information processing [14,15]. The healthy volunteer assay we employed has been widely used, is well-validated, and has been shown to be sensitive to and specific for pharmacological manipulations using conventional antidepressants [15]. In contrast to previous studies on various cognitive effects of pramipexole (e.g., [13,35,36,37,38,39,40]), we used a subacute, rather than an acute dosing scheme including a comparatively high target dose. Compared to a single-dose challenge, subacute pramipexole treatment has higher clinical-ecological validity—especially considering that acute versus subacute dopaminergic challenges likely differ in their neuromolecular effects [8,10,16,41,42].

In addition to the above-mentioned strengths, there are also some limitations of our study that need to be addressed. 

First, blinding to treatment was not fully achieved, as participants and researchers were able to correctly guess treatment allocation to a certain extent. Researchers designing future studies with pramipexole might want to consider this and account for treatment expectations by taking a measure of subjective expected treatment effects and adding these as parametric modulators or covariates to the analysis.

One possible explanation for the lack of full blinding is probably the frequent experience of nausea in the pramipexole group. Yet, despite the high prevalence of this side effect in those receiving pramipexole, it did not give rise to any dropouts. Overall, only two participants decided to leave the study prior to its conclusion because of subjective side effects, but both these participants received placebo (and were replaced by new participants after dropping out).

Second, we tested only healthy volunteers in a certain age range and without a personal or family history of mental health problems. Whilst this allows for a clearer assessment of cognitive effects, it limits the generalisability of results. Future studies might want to also probe the effects of pramipexole on emotional information processing in clinical populations to address this shortcoming.

Third, we did not measure pramipexole serum concentration or prolactin [43] to ascertain compliance with drug intake; however, the high prevalence of nausea in the pramipexole group strongly suggests that most participants adhered to their allocated study treatment.

A final limitation that should be considered is that this present study did not consider the potential effect of ethnicity on emotional processing. Ethnicity (both in terms of participant ethnicity as well as stimulus ethnicity) could be a moderator in accurately identifying facial expressions [44]. Future studies should take this into account.

In conclusion, this randomised, placebo-controlled, experimental medicine study of subacute pramipexole treatment found a valence-specific effect of pramipexole on neural measures of emotional information processing. In line with the cognitive neuropsychological model of antidepressant action, pramipexole treatment led to attenuated bilateral amygdala responses to negative (fearful) relative to positive (happy) facial expressions. However, pramipexole treatment did not induce more positively biased emotional information processing in behavioural tasks. Repeated pramipexole treatment was well tolerated in healthy volunteers, with nausea being the only significant side effect. Taken together, pramipexole’s effects on neural measures of emotional information processing mirror those of conventional antidepressant drugs. Further research is warranted to explore whether this could constitute a relevant mechanism by which pramipexole exerts antidepressant effects.

## 4. Materials and Methods

### 4.1. Participants

Forty healthy volunteers (20 female, 20 male), aged 18 to 43 years, without a personal or family history of any major mental disorder were recruited by advertisement to take part in a larger experimental medicine study that aimed to explore depression-relevant neurocognitive effects of subacute pramipexole treatment. The sample size was based on previously published experimental medicine trials using a comparable design and similar outcome measures and was additionally increased to the maximum number of participants that could be feasibly recruited within the dedicated study period. In an initial screening visit, participants were confirmed to be mentally and physically healthy, as assessed by a structured psychiatric interview (SCID-5) and a general medical interview. In addition, basic demographic, physical, and psychological information was collected (see Table 1). The study was approved by the local ethics board. All study participants gave written informed consent prior to their inclusion in the trial. None of the study volunteers fulfilled any of the pre-specified exclusion criteria (see Table 5).

### 4.2. Study Design and Intervention

**Figure 2:**
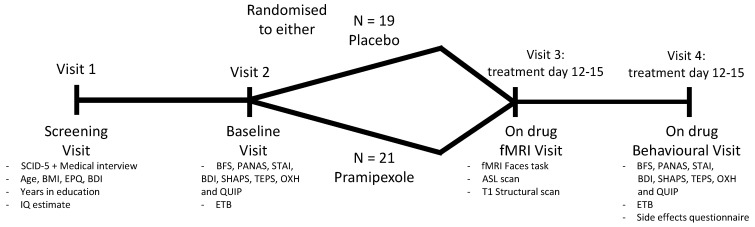
Flowchart describing the study design and intervention. SCID-5: Structured Clinical Interview for DSM-5; BMI: body mass index; EPQ: Eysenck personality questionnaire; BDI: Beck depression inventory; IQ estimate: Spot-the-word test; BFS: Befindlichkeitsskala; PANAS: positive and negative affect schedule; STAI: state-trait anxiety inventory; SHAPS: Snaith-Hamilton pleasure scale; TEPS: Temporal experience of pleasure scale; OXH: Oxford happiness questionnaire; QUIP: questionnaire for impulsive-compulsive disorders in Parkinson’s disease-rating Scale; ETB: Emotional Test Battery; ASL: arterial spin labelling.

The study employed a parallel-group, placebo-controlled, double-blind design (also see Figure 2). All participants were randomly allocated to either placebo (lactose) or pramipexole treatment. Both treatments were administered in indistinguishable capsules. Participants were instructed to take their treatment as a single dose in the evening. The daily dose of pramipexole started at 0.25 mg pramipexole salt and was subsequently increased by 0.25 mg in a stepwise manner every three days until a target dose of 1.0 mg pramipexole salt per day was reached (dose titration based on Fawcett et al. [33]). Participants took the target dose of 1.0 mg for at least two consecutive days before any on-drug assessments were conducted. After all of the testing was completed, pramipexole was tapered down over three days, decreasing the daily dose by 0.25 mg each day. During their participation, all study volunteers could reach a medical doctor 24 h a day via telephone in case of side effects or concerns related to the treatment.

### 4.3. Assessment of Subjective State

Subjective state was assessed once at baseline and once after treatment was established using the following questionnaires: Befindlichkeitsskala of mood and energy (BFS), positive and negative affect schedule (PANAS), state-trait anxiety inventory (STAI), Beck depression inventory (BDI), Snaith–Hamilton pleasure scale (SHAPS), temporal experience of pleasure scale (TEPS), Oxford happiness questionnaire (OXH), and the questionnaire for impulsive-compulsive disorders in Parkinson’s disease-rating scale (QUIP).

### 4.4. Assessment of Side Effects and Blinding

Side effects were assessed on drug (either placebo or pramipexole)using a simple questionnaire asking participants to indicate the presence of a range of potential side effects of pramipexole (see Table 2 for all side effects enquired).

Blinding was checked by means of a forced-choice guess completed by both the study participant and the researcher at the last study visit. 

### 4.5. Assessment of Behavioural Measures of Emotional Information Processing

Emotional information processing was assessed once at baseline and once after the target dose of 1.0 mg pramipexole salt had been established using the tasks described below. Different task stimuli were used for the two assessments.

#### 4.5.1. Facial Expression Recognition Task (FERT)

Participants were presented with pictures of human facial expressions. Each face displayed one of six basic emotions (anger, disgust, fear, happiness, sadness, or surprise). Each emotional expression was presented at different levels of intensity (10%, 20%, 30%, 40%, 50%, 60%, 70%, 80%, 90%, and 100%), which have been created by combining shape and texture features of the two extremes “neutral” (i.e., 0%) and “full prototypical emotion” (i.e., 100%) to varying degrees (based on a previously described procedure by Young et al. [45]). In total, 4 examples of each emotion at each intensity level were presented. Emotions were displayed by 10 different individuals overall, and for each of the 10 individuals, a neutral facial expression was presented as well. Thus, 250 stimulus presentations (6 emotions × 10 intensities × 4 examples + 10 neutral faces) were used in total. Facial expressions were presented in random order on a computer screen for approximately 500 ms each, followed by a blank black screen. Participants were instructed to correctly classify each facial expression as quickly and as accurately as possible. Responses were made by pushing one out of seven labelled buttons on a button box. The main outcomes of interest were hit rate, false alarm rate, non-identification rate, and median reaction time for correct classifications.

#### 4.5.2. Emotional Categorisation Task (ECAT)

Participants were presented with positive and negative personality descriptors and were asked to correctly classify the valence of each word. In total, 40 words describing either extremely agreeable/positive characteristics (e.g., “cheerful”, “honest”, “optimistic”) or extremely disagreeable/negative characteristics (e.g., “domineering”, “untidy”, “hostile”) were presented individually in the centre of the screen for approximately 500 ms each. Positive and negative words were chosen to be comparable with regard to frequency, length, and meaningfulness and were presented in random order. To make the task self-referent, participants were instructed at the beginning to imagine themselves overhearing someone describing them with each of the words indicating as quickly and accurately as possible whether they would like or dislike to be described with each of the words. Responses were made by pressing correspondingly labelled buttons on a button box. The main outcome of interest was the median reaction time for correct classifications.

#### 4.5.3. Emotional Faces Dot Probe Task (FDOT)

Vigilance to emotional stimuli was assessed by comparing behavioural responses to a probe replacing a positive, negative, or neutral emotional cue. Each trial started with the presentation of a fixation cross in the centre of the screen. This was followed by the presentation of a pair of pictures of facial expressions (neutral & neutral, neutral & fearful, or neutral & happy). One face appeared above and the other one below the fixation cross. After approximately 100 milliseconds, both faces disappeared, and two dots in either vertical (:) or horizontal (..) orientation appeared behind one of the faces. Participants were asked to indicate as quickly and as accurately as possible which orientation the dots were in. Half of all trials were masked, i.e., faces were presented on the screen for approximately 16 ms and were then replaced by a jumbled face for approximately 84 ms. The other half of trials were unmasked, with faces simply being presented on the screen for approximately 100 ms. For neutral-emotional pairs, the emotional expression appeared equally often on top and below the fixation cross, and the probe appeared equally often behind the emotional and behind the neutral face. In total, the task consisted of 192 trials (32 happy & neutral, 32 fearful & neutral, 32 neutral & neutral, once masked and once unmasked). The main outcome of interest was the vigilance bias score for emotional stimuli. Vigilance bias scores were calculated for emotional pairs by subtracting median reaction times in congruent trials (i.e., the probe appeared behind the emotional expression) from those in incongruent trials (i.e., the probe appeared behind the neutral expression). If a participant showed, for example, an attentional bias towards positive emotional information, one would expect that, when presented with a happy & neutral face pair, they would identify the probe in congruent trials (probe in the position of the positive face) faster than in incongruent trials (probe in the position of the neutral expression), thus resulting in a vigilance bias score for positive information greater than 0.

#### 4.5.4. Emotional Recall Task (EREC)

Following a distraction period (approximately 15 min of engagement in the FDOT), subjects were asked to recall as many words as possible from the emotional categorisation task. They were given 4 min to write down as many words as they could. The main outcomes of interest were the numbers of correctly and incorrectly recalled words.

### 4.6. Assessment of Neural Activity during Emotional Information Processing

#### 4.6.1. MRI Faces Task

The MRI faces task was designed to investigate the neural response to emotional cues, specifically faces with fearful and happy expressions. The task is known to reliably elicit amygdala activity and has proven to be sensitive to (sub-) acute antidepressant treatment [15,22]. The version of the task presented here has been modified from previous studies with block lengths and orders optimised for current fMRI sequences. 

The stimuli used are colour photographs of fearful and happy faces adapted from the Karolinska directed emotional faces (KDEF) database. Models with unclear expressions of fear or happiness were not included. Participants were instructed that the aim of the task is to correctly identify the gender of the model shown in each image (i.e., male or female) as quickly and as accurately as possible, with no specific reference made to the emotions depicted. Each trial began with the presentation of a fixation cross (displayed for a variable duration (2.7 to 3.1 s)), followed by a face, which was presented in isolation for 100 ms. The task followed an A-B-Rest design, where a block of condition A (fearful faces, 18 s) was followed by a block of condition B (happy faces, 18 s), which was followed by a block of rest (12 s). There was an additional rest block at the start of the experimental run. In total, there were seven repeats of each block, thus, generating 112 s of each emotion condition and 96 s of rest. Each block consisted of six trials in which three male and three female faces were presented in random order. Each image was shown only once over the length of the whole task (3 models per gender over 7 blocks = 42 pictures). A custom Matlab script was used to randomly select images to be used in each block, ensuring that there was an equal number of male and female faces, that each image was used only once, and that the order of presentation was randomised.

#### 4.6.2. Neuroimaging Protocol

Scanning was performed at the Oxford Centre for Human Brain Activity (OHBA), University of Oxford, using a 3-Tesla Siemens Prisma scanner with a 32-channel head-coil. The neuroimaging protocol comprised functional and structural sequences as follows.

##### fMRI—Faces Task

Functional imaging consisted of 60 T2-weighted echoplanar imaging (EPI) slices (TR = 800 ms, TE = 30 ms, flip angle = 52°, field of view = 216 mm, voxel size = 2.4 mm × 2.4 mm × 2.4 mm, acquisition time: 7 min 36 s, multiband acceleration factor = 6 interleaved). Images were distortion-corrected by an acquired fieldmap (echos at 4.92 and 7.38 ms, TR = 482 ms, flip angle = 46°).

##### Structural

In addition, structural scans were acquired via T1-weighted MR images (TR = 1900 ms, TE = 3.97 ms, flip angle = 8°, field of view = 192mm, voxel dimension = 1 mm isotropic, acquisition time: 5 min 31 s). 

##### Arterial Spin Labelling (ASL)

Whole-brain perfusion imaging was performed by using a pseudo-continuous arterial spin labelling (PCASL) sequence, with multi post-labelling delays (at 250 ms, 500 ms, 750 ms, 1000 ms, 1250 ms, 1500 ms), a 2D gradient spin echo readout and a PICORE Q2T labelling scheme. ASL data were collected as tag-control pairs with a TI of 1.8 s and a bolus duration of 1.4 s. TR = 4100 ms, TE = 14 ms, FA 90°, field of view = 220 × 220 mm^2^, 24 slices, voxel dimensions = 3.4 mm × 3.4 mm × 4.5 mm, 97 repeats, acquisition time: 6 min 39 s, fat saturation. A calibration image was acquired but without labelling (TR = 6000 ms). The labelling plane was set with a time of flight neck scan (TR = 21 ms, TE = 3.43 ms, flip angle = 30°, field of view = 200 mm, voxel dimension = 0.3 mm × 0.3 mm × 1.3 mm, acquisition time: 42 s). Images were distortion-corrected by an acquired fieldmap (echos at 4.92 and 7.38 ms, TR = 482 ms, flip angle = 46°).

### 4.7. Data Analysis

#### 4.7.1. Analysis of Questionnaire and Behavioural Data

Analysis of the questionnaire and behavioural data were conducted in SPSS (version 25.0). Subjective state measures were compared between groups using one-way ANCOVAs with post-treatment scores as the dependent variable, group as the independent variable, and pre-treatment score as the covariate. Emotional information processing was analysed using a three-way mixed ANOVA with group as the between-subjects factor and visit and emotion/valence as within-subjects factors, respectively. Effects of interest were group × visit interactions and group × emotion × visit interactions. Where the assumption of sphericity was broken, Greenhouse-Geisser correction was used. In the facial expression recognition task, scores for neutral faces were analysed using the same approach as for the subjective state measures. Side effects and randomization guesses were compared between groups using Fisher’s exact test.

#### 4.7.2. MRI Data Analysis

MRI data were analysed using FSL (FMRIB Software Library v6.0) tools. 

##### fMRI—Faces Task

Structural anatomical scans were brain extracted using Brain Extraction Tool (BET) [46]. Functional MRI data were pre-processed and decomposed into independent components using MELODIC (multivariate exploratory linear optimized decomposition into independent components), part of FSL. Pre-processing involved a number of steps designed to reduce unwanted variability in the data and to improve the validity of the statistical analysis. The following steps were implemented for each participant: (1) Removal of non-brain structures using BET [46], (2) motion correction using MCFLIRT [47], (3) spatial smoothing using a Gaussian kernel of FWHM 5 mm, (4) grand-mean intensity normalisation of the entire 4D dataset by a single multiplicative factor and high-pass temporal filtering cut-off = 90 s (Gaussian-weighted least-squares straight-line fitting, with sigma = 45 s), and (5) B0 unwarping using fieldmap rads and magnitude images for distortion correction.

Functional data were then denoised (removal of noise due to movement, scanner, or cardiovascular artifacts) by independent component analysis (ICA) denoising using FIX, via manually creating a training dataset from 6 subjects in each group (*n* = 12) selected at random. Components were manually labelled by MM; components were retained in cases where it was not clear whether they represented noise.

A custom 3-column format convolved with a gamma hemodynamic response function and its temporal derivative was used to model the data in FEAT (fMRI Expert Analysis Tool—part of FSL). The main contrast of interest was fear versus happy, but fear and happy versus baseline, and the mean of fear and happy versus baseline were also obtained. 

In addition, functional images were registered to their high-resolution structural scan via the high contrast functional image and BBR (Boundary-Based Registration) using FLIRT (FMRIB’s Linear Image Registration Tool) [47,48]. Non-linear registration from structural to MNI standard space was then further refined using FNIRT FMRIB’s Non-Linear Image Registration Tool [49,50], resampling resolution = 2 mm. These transformations into standard space were applied to images of contrasts of interest and their variances. 

Higher level (group level) analysis was carried out using FSL’s tool for nonparametric permutation inference Randomise (5000 permutations) [51], to assess general effects of task-relevant contrasts on both groups, as well as test for group differences. Statistics were assessed using the threshold-free cluster enhancement method with family-wise error correction of 0.05 (or 0.95 threshold within randomise) [52]. The general linear model (GLM) included 2 groups: placebo and pramipexole. Contrasts were defined as placebo greater than pramipexole, pramipexole greater than placebo, and the mean across both groups. Perfusion maps (registered to standard space, smoothed to match the intrinsic smoothness of the rs-fMRI data, voxel-wise demeaned across all subjects, also see section below), as well as a covariate for gender, were added as confounding regressors (nuisance) to the GLM. Given our a priori interest in investigating the effects of pramipexole on the amygdala, small volume correction (SVC) analyses with this region of interest (ROIs) were performed. Anatomical ROIs were generated from the probabilistic maps provided by the Harvard-Oxford Structural Atlas in FSL, threshold = 50 (at least 50% of the voxel belongs to the structure specified). 

Significant brain areas were extracted for visualization using the fslmaths and cluster tool, with a threshold of 0.95 (based on 1/p thresholding from randomise) for the ROI analysis. To further visualise the results, individual parameter estimate (PE) values were extracted from their custom maps, using significant clusters as binary masks. 

All activations are reported using MNI coordinates.

##### Arterial Spin Labelling/Resting Brain Perfusion

Distortion- and motion-corrected resting perfusion maps in units of mL/100g/min were calculated using Oxford_ASL (part of the Bayesian Inference for Arterial Spin Labelling (BASIL) tool, [53]) for each participant, which performs label-control subtraction, inference of voxel-wise perfusion, and voxel-wise calibration to obtain absolute perfusion maps, and controls for partial volume effects at the single-subject level. FSL’s anatomical processing script (FSL_Anat) was used to pre-process each participant’s high-resolution T1 structural image (includes bias-field correction, brain extraction and registration to standard space via FMRIB’s linear image registration tool (FLIRT) and FMRIB’s non-linear image registration tool (FNIRT). The processed perfusion images were non-linearly aligned with standard space via an initial linear transformation T1 structural space (using FLIRT), followed by the application of the non-linear warp from fsl_anat. A Gaussian smoothing kernel of 2.35 mm was applied to all the normalised images (to match functional data). Data were interrogated using a voxel-wise generalized linear model (GLM) permutation nonparametric testing (5000 permutations) with Randomise (FSL’s tool for nonparametric permutation inference on neuroimaging data) [49], correcting for multiple comparisons across space (cluster-based thresholding using TFCE and a family-wise error-corrected cluster significance threshold of *p* < 0.05 applied to the supra-threshold clusters) [51]. This results in whole-brain spatial maps characterising the between-subject/group differences in perfusion (regional perfusion). The GLM comparison included the group of interest comparison (placebo greater than pramipexole and pramipexole greater than placebo). 

Given our a priori interest in investigating the effects of pramipexole on the amygdala, small volume correction (SVC) analyses with this region of interest (ROIs) were also performed, identical to the task fMRI analysis.

##### Global Perfusion

Global grey matter perfusion was defined as mean grey matter perfusion and calculated by thresholding grey matter partial volume maps in ASL native space at 0.80 (taking the average perfusion in voxels with more than 80% grey matter). Treatment groups were compared using *t*-tests in SPSS.

## Figures and Tables

**Table 1:** Basic demographic, physical, and psychological characteristics of 40 healthy volunteers randomly allocated to subacute treatment with either placebo or pramipexole (means with standard deviations in parentheses).

	Placebo (*n* = 19; 10 Male)	Pramipexole (*n* = 21; 10 Male)
Age	24.5 (6.9)	22.5 (3.7)
Body mass index	24.0 (2.9)	22.4 (2.6)
Years in full-time education	17.5 (3.1)	16.8 (2.9)
IQ estimate (spot-the-word test)	111.9 (7.6)	108.3 (8.1)
Neuroticism (Eysenck personality questionnaire)	4.3 (3.7)	4.2 (3.7)
Psychoticism (Eysenck personality questionnaire)	2.8 (2.1)	2.5 (2.1)
Extraversion (Eysenck personality questionnaire)	14.5 (3.7)	14.7 (4.5)
Lie (Eysenck personality questionnaire)	7.5 (3.4)	9.5 (4.6)
Trait anxiety (state-trait anxiety inventory)	32.1 (9.1)	31.2 (9.1)
Depression at inclusion (Beck depression inventory)	2.5 (4.0)	1.6 (1.7)

**Table 2:** Self-reported side effects in the placebo and in the pramipexole treatment group.

	Placebo	Pramipexole	Fisher’s Exact Test (Two-Sided)
Sleeping problems	26.3%	33.3%	*p* = 0.74
Abnormal dreaming	21.1%	19%	*p* = 1.00
Headache	21.1%	23.8%	*p* = 1.00
Dizziness	10.5%	33.3%	*p* = 0.13
Somnolence	21.1%	45%	*p* = 0.18
Nausea	5.3%	61.9%	*p* < 0.001
Vomiting	0.0%	19%	*p* = 0.11
Constipation	5.3%	4.8%	*p* = 1.00
Fatigue	21.1%	23.8%	*p* = 1.00
Impulse control problems	5.3%	9.5%	*p* = 1.00
Hallucinations	0.0%	0.0%	-
Abnormal movements	0.0%	4.8%	*p* = 1.00

**Table 3:** Subjective state scores before and after subacute treatment with either placebo or pramipexole. Values in each group represent means with standard deviations in parentheses. The column on the right-hand side displays ANCOVA results.

	Placebo		Pramipexole		
	Baseline	On-Drug	Baseline	On-Drug	ANCOVA Results
BFS	13.8 (11.2)	14.8 (14.2)	11.7 (12.4)	15.4 (14.7)	F(1.37) = 0.4, *p* = 0.51
STAI state	28.6 (6.1)	29.1 (5.2)	28.9 (6.5)	27.8 (5.4)	F(1.37) = 1.2, *p* = 0.28
BDI	2.8 (3.3)	2.7 (3.3)	1.9 (2.5)	2.9 (3.4)	F(1.36) = 0.9, *p* = 0.36
PANAS pos. present	32.5 (8.6)	32.6 (7.9)	36.4 (7.7)	34.5 (8.3)	F(1.37) = 0.5, *p* = 0.50
PANAS neg. present	11.3 (1.3)	11.8 (2.7)	11.2 (1.5)	11.0 (1.5)	F(1.37) = 2.1, *p* = 0.15
PANAS pos. today	32.9 (8.2)	32.3 (8.1)	36.8 (8.0)	34.8 (9.1)	F(1.37) = 0.1, *p* = 0.73
PANAS neg. today	11.6 (1.7)	11.8 (2.4)	11.4 (2.0)	11.4 (1.7)	F(1.37) = 0.4, *p* = 0.55
PANAS pos. last week	34.2 (7.6)	34.6 (9.3)	37.7 (9.2)	37.0 (7.7)	F(1.37) < 0.001, *p* > 0.99
PANAS neg. last week	14.1 (4.1)	12.6 (2.7)	13.3 (2.8)	12.7 (3.2)	F(1.37) = 0.1, *p* = 0.71
SHAPS	0.2 (0.7)	0.3 (0.7)	0.5 (1.0)	0.7 (1.6)	F(1.37) = 0.3, *p* = 0.61
TEPS total	79.3 (8.2)	79.2 (10.0)	85.3 (10.0)	82.1 (9.5)	F(1.37) = 0.7, *p* = 0.42
TEPS anticipatory	42.1 (4.9)	42.3 (5.4)	47.1 (6.1)	44.6 (5.5)	F(1.37) = 0.8, *p* = 0.38
TEPS consummatory	37.3 (4.4)	36.8 (5.4)	38.1 (5.6)	37.5 (5.9)	F(1.37) = 0.01, *p* = 0.91
OXH	132.4 (19.6)	132.0 (19.7)	134.8 (19.2)	138.0 (18.4)	F(1.37) = 3.1, *p* = 0.08
QUIP	16.6 (11.2)	13.8 (10.4)	12.3 (8.3)	8.7 (8.5)	F(1.37) = 1.0, *p* = 0.33

BFS: Befindlichkeitsskala; STAI: state-trait anxiety inventory; BDI: Beck depression inventory; PANAS: positive and negative affect schedule; SHAPS: Snaith-Hamilton pleasure scale; TEPS: temporal experience of pleasure scale; OXH: Oxford happiness questionnaire; QUIP: questionnaire for impulsive-compulsive disorders in Parkinson’s disease rating scale.

**Table 4:** Behavioural measures of emotional processing at baseline and on-drug. Values represent means with standard deviations in parentheses.

	Placebo		Pramipexole	
	Baseline	On-Drug	Baseline	On-Drug
Facial expression recognition task (FERT)				
Hit rate (%)				
Anger	55.6 (10.5)	59.3 (7.7)	53.3 (12.7)	57.9 (14.3)
Disgust	66.9 (9.6)	67.5 (12.6)	62.3 (15.8)	62.1 (17.5)
Fear	54.6 (15.2)	61.1 (10.2)	49.4 (17.2)	55.6 (16.2)
Happiness	80.3 (5.4)	78.2 (5.3)	77.2 (7.3)	74.8 (5.5)
Sadness	63.5 (8.5)	65.1 (9.3)	63.2 (12.5)	61.2 (12.3)
Surprise	68.9 (6.5)	74.2 (5.5)	67.4 (8.3)	72.6 (6.8)
Neutral	83.3 (12.4)	90.6 (10.0)	87.6 (12.6)	87.6 (17.6)
False alarm rate (%)				
Anger	1.7 (1.1)	1.8 (1.9)	1.7 (1.9)	2.1 (2.1)
Disgust	2.4 (1.7)	1.9 (1.4)	2.5 (2.0)	2.2 (1.7)
Fear	1.3 (0.9)	1.2 (0.9)	0.9 (1.2)	1.2 (1.2)
Happiness	0.5 (0.8)	0.6 (0.8)	0.5 (0.7)	0.7 (0.8)
Sadness	3.0 (2.4)	2.8 (2.4)	3.5 (3.3)	3.5 (2.7)
Surprise	2.5 (1.9)	1.6 (1.4)	3.4 (2.5)	2.0 (2.1)
Non-identification rate (%)				
Anger	33.8 (7.0)	32.8 (8.3)	35.6 (9.4)	34.3 (11.3)
Disgust	21.7 (5.2)	18.2 (7.4)	24.3 (7.2)	22.1 (8.5)
Fear	23.2 (2.5)	22.5 (4.5)	23.8 (6.1)	22.4 (5.1)
Happiness	16.8 (5.5)	18.6 (5.2)	19.3 (5.9)	21.0 (4.8)
Sadness	32.6 (7.7)	31.8 (8.9)	34.3 (11.9)	35.0 (10.8)
Surprise	25.8 (4.9)	21.0 (5.3)	27.0 (4.7)	23.5 (4.1)
Reaction time (ms)				
Anger	1429.6 (279.2)	1356.6 (231.3)	1396.9 (348.1)	1274.1 (202.4)
Disgust	1435.8 (247.4)	1246.1 (194.0)	1437.1 (307.3)	1245.3 (165.6)
Fear	1645.4 (325.7)	1456.9 (314.7)	1646.9 (446.1)	1443.8 (316.5)
Emotional categorisation task (ECAT)				
Reaction time (ms)				
Positive	724.9 (97.3)	802.4 (120.0)	748.0 (147.5)	771.1 (126.1)
Negative	835.3 (135.0)	869.3 (153.9)	799.0 (138.6)	823.0 (171.1)
Faces dot probe task (FDOT)				
Vigilance bias score (ms)				
Masked positive	−7.7 (38.4)	−11.0 (54.1)	8.1 (33.0)	9.5 (37.5)
Masked negative	−3.4 (43.8)	−2.9 (32.1)	−16.1 (40.5)	−8.0 (28.1)
Unmasked positive	−4.9 (40.5)	4.9 (35.6)	4.0 (34.3)	−1.3 (40.3)
Unmasked negative	−0.1 (46.0)	−10.7 (33.7)	−12.0 (40.0)	−2.4 (31.3)
Emotional recall task (EREC)				
Hits (n correct)				
Positive	4.4 (1.2)	5.6 (2.7)	5.6 (2.5)	6.4 (3.2)
Negative	3.5 (1.0)	3.2 (2.1)	4.0 (2.3)	4.2 (2.2)
Intrusions (n wrong)				
Positive	3.2 (2.8)	7.9 (3.2)	2.4 (2.7)	8.9 (3.8)
Negative	1.5 (1.6)	4.4 (2.2)	1.4 (1.7)	5.1 (2.1)

**Table 5:** Exclusion criteria for the study.

Exclusion Criteria
Current or past psychiatric disorder (e.g., depression, bipolar disorder)
First-degree relative with a diagnosis of schizophrenia-spectrum or other psychotic disorder, or bipolar disorder
Body mass index not between 18 and 30
History of unexplained hallucinations or impulse control problems (e.g., pathological gambling)
Severe medical condition not stabilized at the time of the experiment (e.g., cardiovascular disease, epilepsy, asthma)
Severe heart or blood vessel disease
Postural hypotension
Any history of seizures
Lactose intolerance
Any current or past physical illness that has the potential to significantly affect mental functioning (e.g., epilepsy, hypothyroidism, Parkinson’s disease, multiple sclerosis)
Pregnant or lactating woman
Sexually active woman who does not use any medically accepted method of contraception
Current or previous intake (last three months) of any medication that has a significant potential to affect mental functioning (e.g., benzodiazepines, antidepressants, neuroleptics)
Any intake of recreational drugs in the last 3 months (e.g., marijuana, ecstasy)
Regular alcohol consumption of more than 14 units a week or excessive alcohol consumption up to three days before the experiment
Regular smoker (> 5 cigarettes per day)
Excessive caffeine user (> 6 caffeinated drinks per day)
History of recurrent rashes or history of allergic reactions to relevant substances (e.g., pramipexole)
Previous participation in a study using the same or similar tasks
History of recurrent rashes or history of allergic reactions to relevant substances (e.g., pramipexole)
Previous participation in a study using the same or similar tasks
Any contraindication to magnetic resonance imaging (e.g., metallic implant, severe claustrophobia)
Current participation in another study
In the researcher’s opinion, participation in the study could be harmful or severely distressing to the participant (e.g., intolerance of side effects), or the participant is not able to follow instructions or complete study tasks

## Data Availability

The data presented in this study are available on request from the corresponding author. The data are not publicly available due to disclosure risk concerning sensitive personal information.

## Supplementary Materials

The following are available online at https://www.mdpi.com/article/10.3390/ph14080800/s1, Figure S1: Activation of brain areas during the fMRI emotional faces task.
