# Supplementary material for: An Experimental Medicine Investigation of the Effects of Subacute Pramipexole Treatment on Emotional Information Processing in Healthy Volunteers
Source: Pharmaceuticals (Basel). 2021 Aug 14;14(8):800. doi: 10.3390/ph14080800 (PMC8401454; doi:10.3390/ph14080800)
Supplement: Supplementary file 1 [file pharmaceuticals-14-00800-s001.zip › pharmaceuticals-1260650-supplementary.pdf]

Supplementary results

Neural activity elicited by the fMRI emotional faces task (whole brain analysis)

In order to determine if the fMRI task engaged brain regions previously associated with fearful and happy facial stimuli, blood-oxygen-level-dependent (BOLD) activation in response to fearful faces, happy faces, and the mean of both valences was compared to baseline (i.e., fixation cross) across both groups. Significant brain activations were observed in a network of areas that match previous reports. As illustrated in Figure S1 in red, the task activated expected areas such as left and right temporal occipital fusiform cortex, bilateral occipital cortex, left and right precentral gyrus extending into postcentral gyrus, right middle frontal gyrus, right precentral gyrus extending into inferior frontal gyrus and frontal pole, left central opercular cortex extending into putamen, thalamus, left amygdala, bilateral insular cortex, right frontal orbital cortex and cerebellum. There were also task-elicited deactivations (i.e., significant activation in fixation cross compared to faces, illustrated in blue in Figure S1) in default mode network areas such as medial prefrontal cortex, bilateral temporal poles, posterior and anterior cingulate cortex, parahippocampal gyri and lateral occipital cortex. Together, these findings therefore confirm that the task engages brain regions that are part of a network implicated in the processing of both fear and happiness [22,24,25].

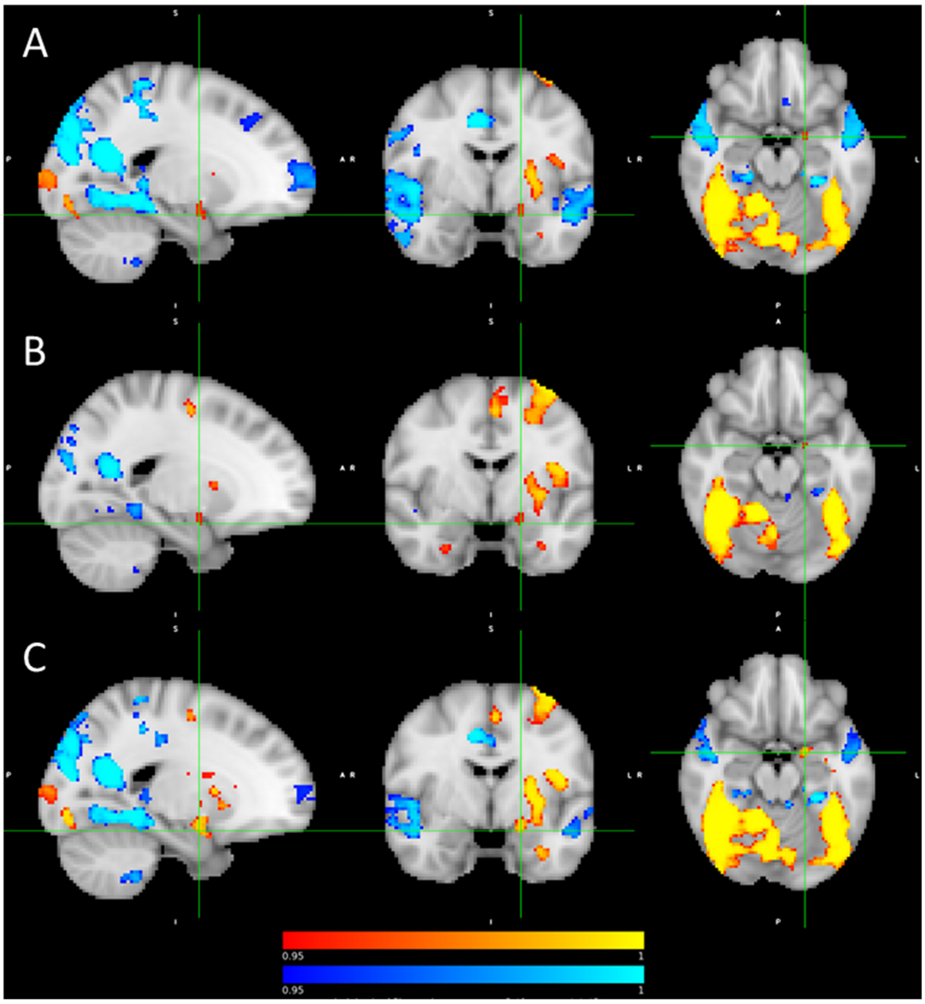

Figure S1: Activation of brain areas during the fMRI emotional faces task. The sagittal, coronal and axial images depict neural activation (red) and deactivation (blue) in response to (A) fearful faces versus fixation cross (B) happy faces versus fixation cross and (C) mean of both valences, across both treatment groups. Cursor in left amygdala. Results are shown TFCE-corrected with a family-wise error cluster significance level of 1 - p > 0.95.
